# *Spiroplasma* in *Glossina fuscipes fuscipes*: a potential manipulator of male reproduction

**DOI:** 10.1128/msystems.00526-26

**Published:** 2026-09-11

**Authors:** Riccardo Piccinno, Giulia Fiorenza, Francesco Lescai, Simone Carpanzano, Fabian Gstöttenmayer, Kiswend-sida M. Dera, Anna Cleta Croce, Chantel J. de Beer, Brian L. Weiss, Mariangela Santorsola, Alberta Pinnola, Giuliano Gasperi, Federico Forneris, Serap Aksoy, Adly M. M. Abd-Alla, Anna Rodolfa Malacrida

**Affiliations:** 1 Department of Biology and Biotechnology “Lazzaro Spallanzani”, University of Pavia 19001, Pavia, Italy; 2 Department of Epidemiology of Microbial Diseases, Yale School of Public Health 50296 , New Haven, Connecticut, USA; 3 Insect Pest Control Laboratory, Joint FAO/IAEA Programme of Nuclear Techniques in Food and Agriculture, Vienna, Austria; 4 Institute of Molecular Genetics (IGM), Italian National Research Council (CNR), Pavia, Italy; 5 Fondazione IRCCS Policlinico San Matteo 320695 , Pavia, Italy; California State University Stanislaus, Turlock, California, USA

**Keywords:** *Spiroplasma*, *Glossina fuscipes fuscipes*, microbiota, male reproduction, energy metabolism, differential gene expression

## Abstract

*Glossina fuscipes fuscipes* (*Gff*) is a riverine species of the *Glossina* genus (tsetse flies). It is one of the principal vector species responsible for transmitting human African trypanosomiasis across a broad swath of sub-Saharan Africa. Consequently, it remains a primary target for vector-control interventions in endemic regions. As with other *Glossina* species, *G. fuscipes* houses a highly streamlined microbiota, composed of maternally transmitted endosymbionts mandatory for survival, which also significantly impacts the vectorial capacity. Among them, the obligate *Wigglesworthia* provides essential nutrients and stimulates maturation of the fly’s immune system, providing a refractory effect against *Trypanosoma*. It also harbors the commensal *Sodalis* and parasitic *Wolbachia*, the latter of which induces cytoplasmic incompatibility. In addition, *Gff* hosts a *Spiroplasma* strain that enhances host refractoriness to trypanosome infection but disrupts female metabolic and reproductive homeostasis. We used high-throughput RNA sequencing to examine the impact of *Spiroplasma* on male testes and accessory glands to reveal how this microbe exerts its influence on male reproductive physiology. We found that *Spiroplasma* substantially alters testes physiology, downregulating genes involved in energy metabolism and sperm function, suggesting reduced male reproductive fitness. In contrast, accessory gland gene expression was not significantly affected despite the critical role of these organs in semen production and reproductive success, considering also that the bacterium exploits the ejaculate to further increase its population prevalence. *Spiroplasma* infection also correlated with increased abundance of other symbionts, including *Wigglesworthia*.

**IMPORTANCE:**

A complex host-symbiont interplay makes the *Spiroplasma* harbored in tsetse fly intriguing. The unexpected male-specific energetic costs of *Spiroplasma* infection keep open the biological question of why this bacterium is maintained in this species. On the other hand, *Spiroplasma* infection correlates with increased abundance of *Wigglesworthia*, promoting trypanosome refractoriness. The present work adds a valuable layer to further interpret the ecological and evolutionary narrative of *Spiroplasma* interactions, and lays the preliminary groundwork to explore the potential of using *Spiroplasma* as a biocontrol agent. While acknowledging that our findings were derived from a long-established laboratory colony, the data on the modulation of fly reproductive fitness and on the possible contribution to trypanosome refractoriness found make *Spiroplasma* intriguing for strategic integration into environmentally sustainable vector management programs, potentially in concert with the Sterile Insect Technique. The results provide significant advances in symbiont-host interactions, bringing new insight into understanding the role of symbionts in viviparous tsetse flies. We also provide a community forum for discussion on the possible use of bacteria as species-specific biocontrol.

## INTRODUCTION

*Glossina fuscipes fuscipes* (*Gff*) is a riverine species of the *Glossina* genus, comprising flies known as tsetse. These flies are medically and economically important vectors of human African trypanosomiasis (HAT), which impart severe epidemiological and socioeconomic consequences, including fatal outcomes in untreated humans (1, 2). *Gff* is one of the principal vector species responsible for transmitting HAT (3) across a broad swath of sub-Saharan Africa. Consequently, it remains a primary target for vector-control interventions in endemic regions (4, 5).

They exhibit peculiar microbiota-dependent biology that significantly impacts their vectorial capacity. During their evolution (6, 7), flies in the genus *Glossina* have developed a variety of specialized traits, including exclusive reliance on vertebrate blood meals by both sexes and an obligate viviparous mode of reproduction. Alongside these adaptations, *Glossina* species have developed intricate relationships with specific microbes ranging from mutualistic to parasitic. These symbiotic relationships provide fundamental physiological support that enables and sustains tsetse’s specialized biology (8–11). Notably, tsetse reproduces via a process called “adenotrophic viviparity,” during which larval offspring develop in the maternal uterus where they are nourished via glandular milk secretions (12). *Glossina* consequently exhibit relatively long gonotrophic cycles (8–11 days), low fecundity, and longer lifespan. These features require a substantial temporal and metabolic investment that leads to the production of a smaller number of offspring (13, 14). Likely because *Glossina* feed exclusively on sterile vertebrate blood, and their larval offspring develop in the maternal uterus, these flies house a highly streamlined microbiota, composed almost exclusively of maternally transmitted endosymbionts, mandatory for survival. All tsetse house obligate *Wigglesworthia* provides essential nutrients missing from vertebrate blood and stimulates maturation of the fly’s immune system during larval development (15). In addition, some tsetse flies harbor commensal *Sodalis* and parasitic *Wolbachia*, the latter of which induces cytoplasmic incompatibility in tsetse (16).

Some laboratory-reared and wild tsetse fly species within the *Palpalis* subgroup were recently found to be infected with another vertically transmitted bacterial endosymbiont from the genus *Spiroplasma* (17, 18). These spirochete bacteria, which lack a cell wall, are commonly associated with arthropods and plants in which they exhibit pathogenic, commensal, or mutualistic associations (19–21). The *Spiroplasma* strain associated with *Gff*, denoted *s*Gff (22; NCBI BioProject: PRJNA1235259), falls within the Citri clade of the Poulsonii group (17, 23). This strain, by contrast to *S. poulsonii* in *Drosophila melanogaster*, does not exhibit a male-killing phenotype (19), but is able to disrupt *Gff* metabolic and reproductive homeostasis (24). However, *s*Gff is able to render *Gff* flies refractory to trypanosome infections (18). These characteristics make this bacterium an intriguing potential tool for enhancing disease control methods. *s*Gff can be transmitted vertically from mother to larvae and horizontally from an infected male to an uninfected female during mating (23, 24). Horizontal transmission from males to females, and the possible subsequent vertical transmission of the endosymbiont to progeny, happens via the infected ejaculate enclosed within the spermatophore. After mating, the spermatophore dissolves and its contents, including the sperm bundle and sGff, migrate to the spermathecae, where they are stored (23). Therefore, this bacterium may exploit maternal and paternal transmission modes to spread and be maintained within natural populations.

*s*Gff impacts *Gff* mating behavior and reproductive physiology. More specifically, the bacterium promotes premating isolation, leading to nonrandom mating patterns that favor the pairing of individuals with matching infection statu*s* (23). sGff infection also impacts its host’s reproductive physiology by lengthening *Gff*’s gonotrophic cycle and reducing the motility of spermatozoa stored in the female spermathecae. These phenotypes result from competition between the fly and bacterium for a finite supply of lipids that are metabolically critical for both organisms (24–26). Whether these *s*Gff-induced phenotypes contribute to shaping the prevalence of infected flies in wild populations remains an open question. Also unknown is whether these phenotypes contribute to the *Gff* differentiation observed in Uganda (27).

The potential interactions between *s*Gff and other resident microbiota are another important aspect to be considered. Previous studies have reported that tsetse fly species within the Palpalis group, which host *Spiroplasma* infections, have significantly lower *Wolbachia* infection prevalence and density than fly species within the Morsitans subgroup that do not house *Spiroplasma* (17). However, interactions between *Spiroplasma* and other tsetse endosymbionts still require elucidation.

*s*Gff exhibits a broad tissue tropism in *Gff*. The bacterium has been detected in midguts, heads, hemolymph, and the female (ovaries, spermathecae) and male (testes, in which it presents the highest density, and male accessory glands [MAGs]) reproductive tracts (17, 25). However, the potential impact of *s*Gff on male metabolic and reproductive physiology in the male reproductive tract remains largely unknown. Herein, we employed high-throughput RNA sequencing on testes and MAGs of sGff-infected and -uninfected males to better understand the mechanisms by which the bacterium may modify host responses in these tissues. Such an approach offered a molecular perspective on how this symbiont negatively affects the testes’ key reproductive processes, such as mitochondrial dynamics, energy metabolism, and sperm functionality. On the other hand, this bacterium does not seem to impact the functionality of male accessory glands, which are involved in semen production, a critical component for male reproductive success, despite using the ejaculate as a way of transmission.

## RESULTS

### Gene expression profiles in testes and male accessory glands

From a heterogeneously infected *Gff* laboratory strain, 24 pooled samples of testes and accessory glands have been prepared. Flies’ infection status was assessed by screening adult legs through PCR prior to pooling. The samples included six pools of testes and six pools of MAGs derived from *s*Gff-infected males, and six pools of testes and six pools of MAGs from uninfected males. A total of 1.59 billion high-quality reads were obtained (66.05 million reads per sample on average). Each pool contained tissues collected from 10 males. No significant difference in the number of mapped reads between MAGs and testes (*U* = 95, *n*1 = *n*2 =12, *P* = 0.198; Mann-Whitney *U* test) and between positive and negative (*U* = 96, *n*1 = *n*2 =12, *P* = 0.172; Mann-Whitney *U* test) was observed.

To have a picture of the gene expression profile of *Gff* testes and MAGs, only *s*Gff-uninfected samples were considered. A total of 10,040 transcribed genes were identified across the 12 uninfected samples (Table S1).

**Fig 1:**
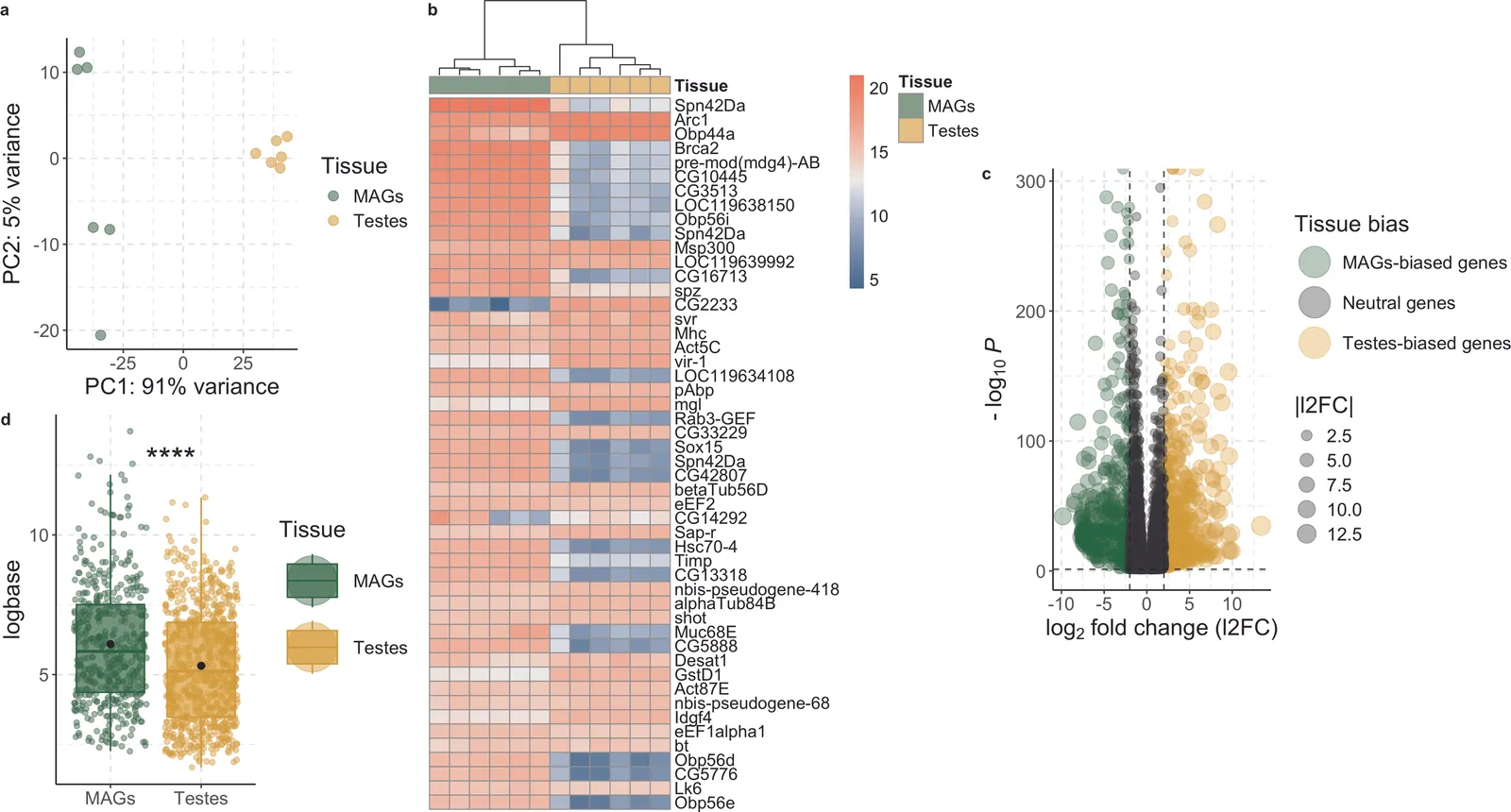
Gene expression differences between *Glossina fuscipes fuscipes* testes and MAGs. (**a**) PCA plot considering the first two principal components calculated on the normalized counts of *s*Gff-uninfected samples. PC1 is the major component distinguishing the two tissues, which explains the 91% variance between testes and MAGs. (**b**) Heatmap of the 50 highly expressed genes across uninfected samples. When available, *Drosophila melanogaster* orthologs are shown to the right of each row; otherwise, the original *Gff* gene symbols are retained. Colors range from blue (lower expression) to red (higher expression) and represent sample-normalized counts for each gene. The heatmap highlights that MAGs are generally characterized by higher transcriptional activity compared to testes. (**c**) Volcano plot showing differentially expressed genes (DEGs) of *s*Gff-uninfected samples (|l2FC| ≥ 2 and *P*adj ≤ 0.05). (**d**) Boxplot comparing the gene transcription (normalized read counts) of differentially expressed genes between tissues, considering uninfected samples, which are significantly different (*P* = 7.06e^−10^; ****). The two tissues’ black points define the mean of the two groups (MAGs = 6.09, testes = 5.31). Jitters show genes significantly differentially expressed and biased toward two tissues, with more in testes (*n* = 739) compared to MAGs (*n* = 473). *Gff, Glossina fuscipes fuscipes*; MAGs, male accessory glands; PC, principal component; *P, P*-value; *P*adj, adjusted *P*-value; l2FC, log2 fold change.

Principal component analysis (PCA) applied to the 12 uninfected samples shows a clear-cut division between the expression of testes and MAG genes, respectively (Fig. 1a). Most of the gene expression variance is represented in the first principal component (PC1: 91%), by which the two reproductive tissues are clearly separated. Genes were then sorted by their mean expression level across the uninfected samples, and clustering was performed based on the sample-wise gene expression levels of the first 50 genes. The derived heatmap of gene expression profiles of these genes in these two tissues shows a smaller number of expressed genes but an overall greater expression level in MAGs with respect to testes (Fig. 1b).

Differential gene expression analysis between *s*Gff-uninfected MAG and testes reported 1,173 DEGs (|log2 fold change [l2FC]| ≥ 2 and *P* ≤ 0.05; Fig. 1c; Table S1). Of these, 689 DEGs were upregulated and 484 downregulated in testes compared to MAGs (Fig. 1c). The difference in DEG expression level between MAGs (MAG-biased DEGs; mean = 6.09, standard deviation [sd] = 2.17) and testes (testis-biased DEGs; mean = 5.31, sd = 2.04) is significant (*t*_960_ = 6.23, *P* = 7.06e^−10^; Fig. 1d).

An over-representation analysis (ORA), considering all 1,173 DEGs, indicated that 22 molecular functions (MFs) and 32 biological processes (BPs) were significantly influenced by testis-biased DEGs, whereas 28 MF and 35 BP were found significantly affected by MAG-biased DEGs (Fig. S1 and S2; Table S2). Focusing on the most represented categories, the top three testis-biased BP were oxidoreductase activity, protein-hormone receptor activity, and metallocarboxypeptidase activity. In contrast, the top MAG-biased BP categories were procollagen-prolyl 4-hydroxylases and DNA-binding transcription factor activity. For MF, the most represented categories for testes were transmembrane transport, oligosaccharide biosynthetic process, and glutathione metabolic process, while the top category for the MAGs was the mannose metabolic process.

### *s*Gff impacts on gene expression in testes and MAGs

**Fig 2:**
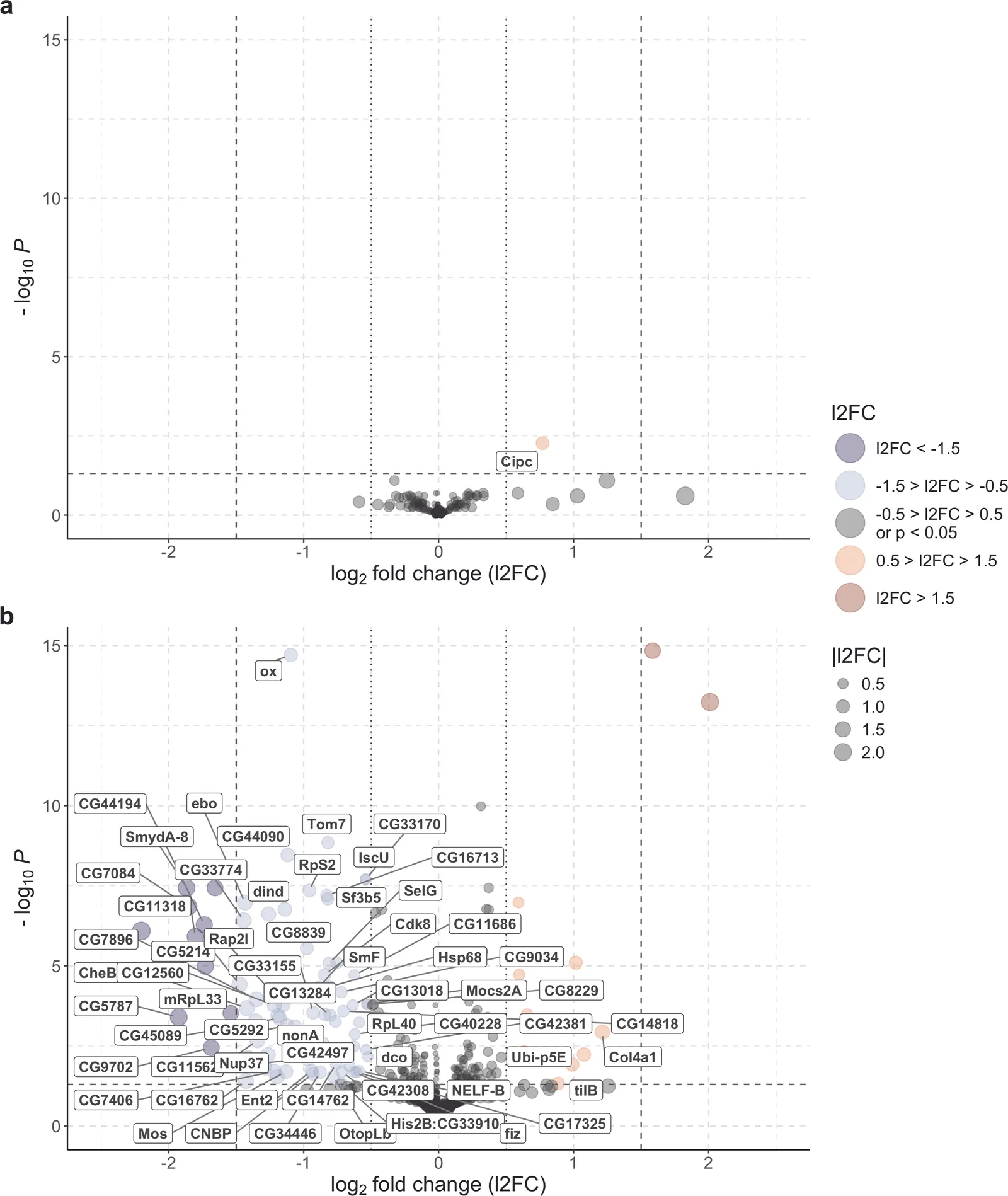
Volcano plot showing differentially expressed genes in *s*Gff-infected and *s*Gff-uninfected MAGs (**a**) and testes (**b**). Genes with l2FC ≥1.5 and adjusted *P* ≤ 0.05 are significantly upregulated in *s*Gff-infected samples (red), while those with l2FC ≥0.5 and adjusted *P* ≤0.05 are moderately upregulated (light red). Conversely, genes with l2FC ≤−1.5 and adjusted *P* ≤0.05 are significantly upregulated in *s*Gff-uninfected samples (blue), while those with l2FC <−0.5 and adjusted *P*-value <0.05 are moderately upregulated (light blue). Genes with −1.5 < l2 FC < 1.5 and adjusted *P*-value >0.05 are not significantly differentially expressed (black). Dashed black lines indicate thresholds for significance (−log₁₀*P* = 1.3, |l2FC| = 1.5), and dotted black lines indicate |l2FC| = 0.5. Point size corresponds to the magnitude of |l2FC|, and differentially expressed genes with *Drosophila melanogaster* orthologs are labeled. l2FC, log2 fold change; *P*, adjusted *P*-value; MAGs, male accessory glands.

A single DEG (|l2FC| ≥ 0.5 and *P* ≤ 0.05), corresponding to *Drosophila* ortholog *Cipc* (Clock Interacting Protein Circadian), was found significantly upregulated out of 9,408 transcribed genes obtained from the comparison between infected and uninfected MAGs (*P* = 0.005, l2FC = 0.77; Fig. 2a; Table S3). No downregulated genes were found. Conversely, 88 significant DEGs (|l2FC| ≥ 0.5 and *P* ≤ 0.05) were identified between positive and negative testes out of 10,151 transcribed genes (Fig. 2b; Table S4).

Of the 88 testes DEGs (73 of which have a *D. melanogaster* ortholog), 12 were classified as pseudogenes (5 downregulated and 7 upregulated), 71 (59 with *D. melanogaster* orthologs) were downregulated, and only 5 (3 with *D. melanogaster* orthologs) were upregulated. Among them, *ctenidin-3-like* (downregulated) (28), antimicrobial; *tilB* (upregulated) and *atk* (downregulated), involved in flagellar function (29, 30); *ox* (downregulated), central component of the cytochrome c oxidase (31); *Gld* (downregulated), coding for the glucose dehydrogenase; and *Ubi-p5e* (upregulated) and *RpL40* (downregulated) involved in ubiquitin activity (32).

### Biological processes affected by sGff in Gff testes

**Fig 3:**
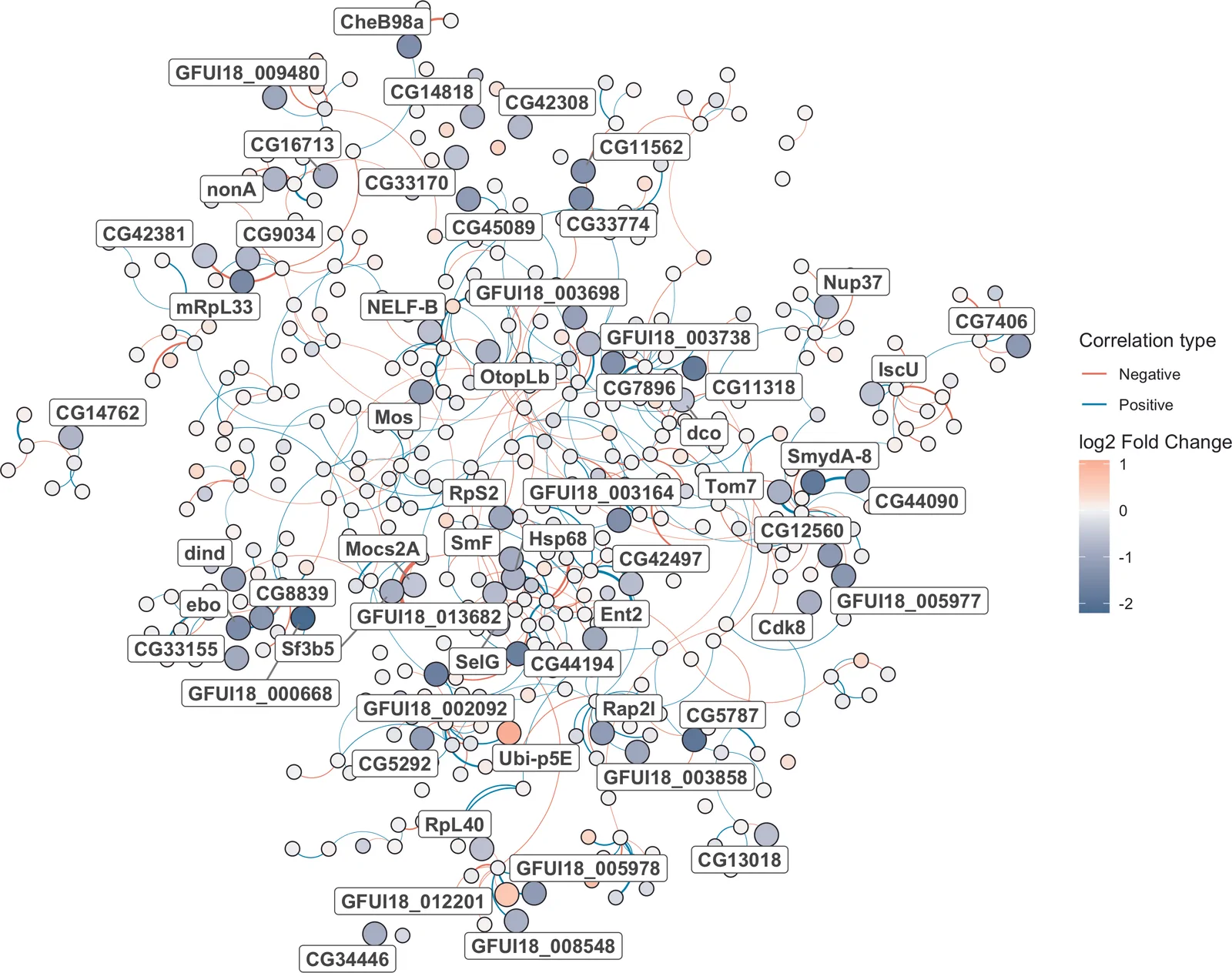
Network graph generated from the co-expression network analysis. Each point represents a gene that belongs to the “yellow” co-expression module (comprising 523 out of 10,151 genes), identified using an unsigned topological overlap matrix (TOM). This module contains most of the differentially expressed genes (59/88 genes; reported in labels and greater node size). The network visualization displays the minimum spanning tree computed from the Pearson correlation adjacency matrix of the module’s TOM. Node colors range from blue (downregulated in *s*Gff-infected testes) to red (upregulated in *s*Gff-uninfected testes), passing through white (neutral). Edge color represents the type of correlation (positive is red and negative is blue). The greater the correlation between nodes, the thicker the edges.

An ORA considering the 88 DEGs between infected and uninfected testes identified five downregulated biological processes: anion transport, inorganic anion transport, sulfate transport, sulfur compound transport, and mitochondrial cytochrome c oxidase (Fig. S3). A co-expression analysis of the 10,151 transcribed genes in testes was performed using WGCNA (soft-thresholding power = 6, minimum module size = 30, and merge cut height = 0.25). This analysis revealed that 59 of the 88 DEGs are part of the same co-expression network, characterized by 523 genes belonging to the “yellow” module (Fig. 3). This co-expression network defines the overall mitochondrial activity, including biological processes such as mitochondrial respiratory chain complex assembly, cytochrome complex assembly, cellular components such as mitochondrial respirasome, mitochondrial respiratory chain complex IV, ribonucleoprotein complex, ribosome, and molecular functions such as structural constituent of ribosome (Table S5).

**Fig 4:**
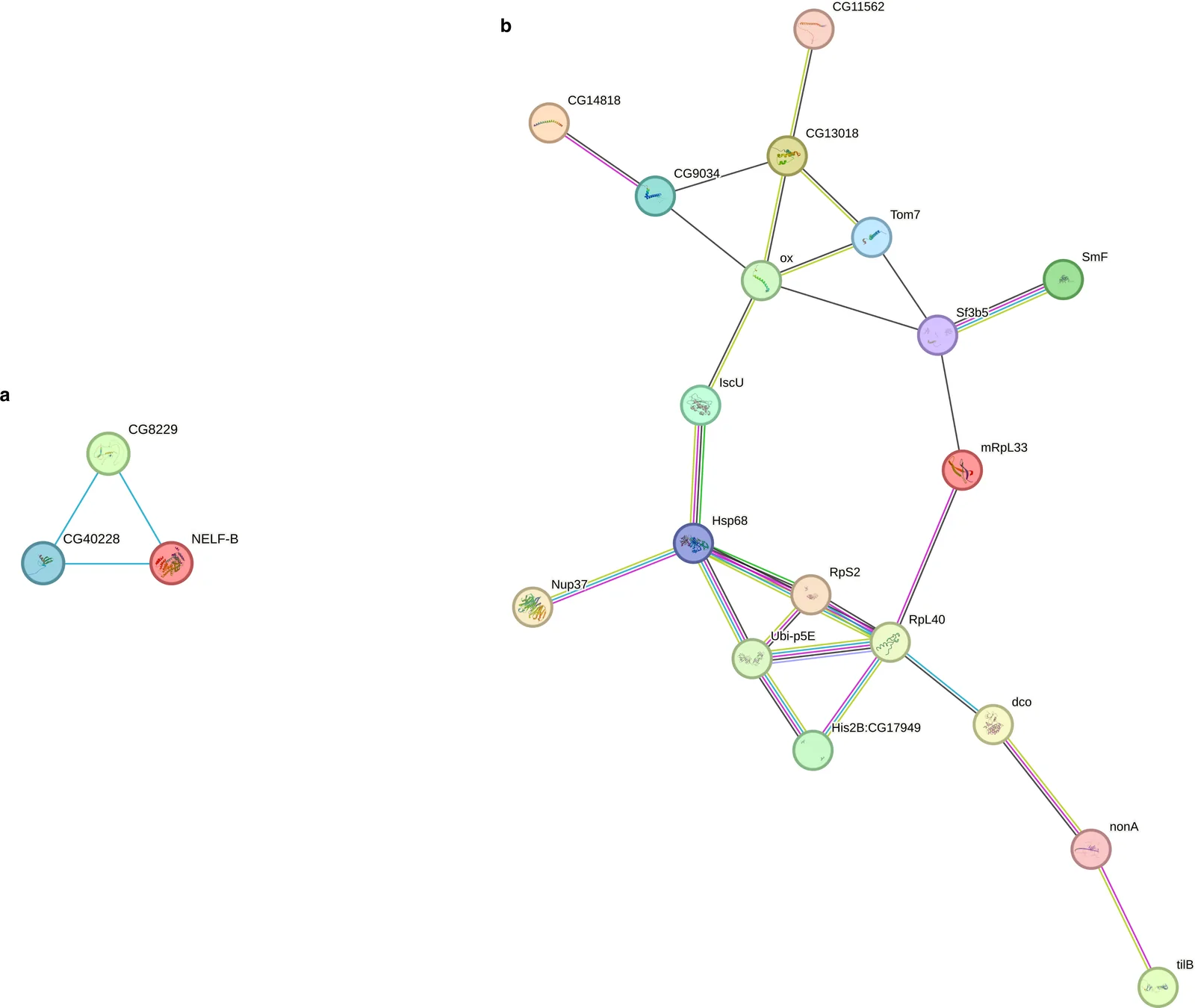
STRING results considering the 73 DEGs having a *D. melanogaster* ortholog. Twenty-one of them were linked to each other in a network. (**a**) A network of 21 interconnected DEGs. (**b**) A separate network of three DEGs. Nodes represent DEGs, while edges indicate interactions classified by color: blue for known interactions from curated databases, pink for experimentally determined interactions, light green for predicted gene neighborhood associations, yellow-green for text-mined interactions, and black for predicted co-expression.

The 73 out of the 88 DEGs having a *D. melanogaster* ortholog were submitted to STRING (33) and originated two connected networks. The smallest network was composed of three genes (Fig. 4b) and was associated with the transcription elongation factor complex (CL:7140, STRING CLUSTER db). The other network was composed of 21 genes (Fig. 4a), and it was associated with three KEGG and 123 Reactome pathways (Table S6). Most of the Reactome results were due to the presence of *RpL40* and *Ubi-p5E* in the network, which are related to ubiquitin activity (32).

### *s*Gff impact on sperm mitochondrial activity

**Fig 5:**
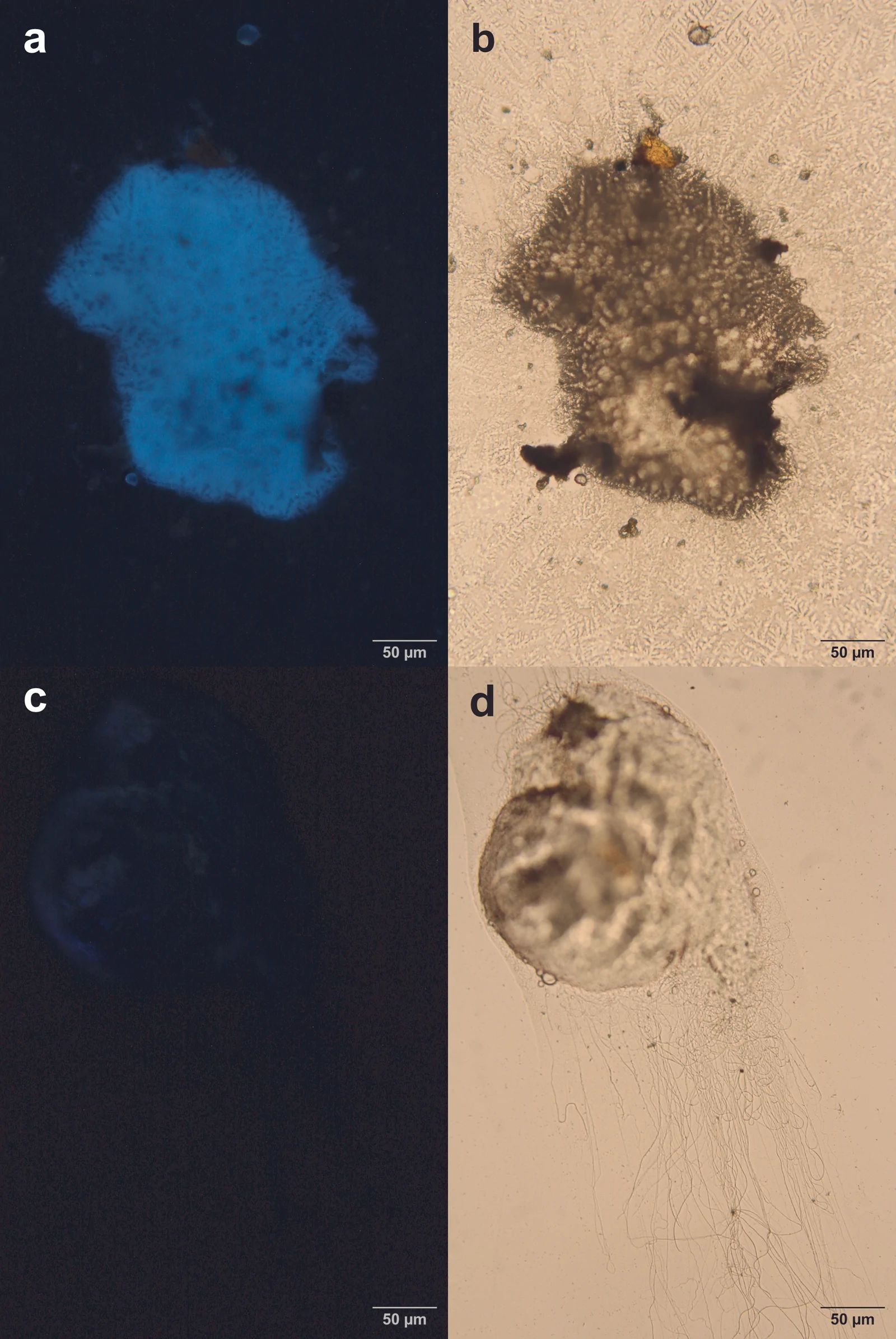
(**a**) *s*Gff-uninfected sperm bundle in fluorescent light, (**b**) *s*Gff-uninfected sperm bundle under microscope, (**c**) *s*Gff-infected sperm bundle, and (**d**) *s*Gff-infected sperm bundle under microscope.

To directly evaluate the energy availability in sperm, we quantified the intrinsic autofluorescence (AF) of NADH. Since only the reduced form of this coenzyme is naturally fluorescent, its signal intensity serves as a reliable, label-free proxy for mitochondrial activity and cellular energy levels (34). The autofluorescence spectra collected from both spermatophores and the sperm bundle, isolated from spermatophores, covered the 400–600 nm spectral range, with a main band in the 400–550 nm region, largely attributable to NAD(P)H emission. When normalized to 100 a.u. at the maximum peak, the emission spectral profiles from *s*Gff-infected or *s*Gff-uninfected spermatophores did not show remarkable differences (Fig. S4; Table 1). By contrast, significant differences in sperm bundle autofluorescence have been observed between infected and uninfected sperm (*P* = 0.01; Table 1). Uninfected sperm displayed a slightly higher tail in the yellow-red region (520–650 nm; Fig. S4), ascribable to a minor contribution from fluorescing species different from NAD(P)H (Fig. 5a and b), whereas infected sperm exhibited a shift of about 10 nm toward the blue region (Fig. S4), suggesting a shift from the free state of mitochondrial NADH toward the bound state with higher fluorescence (Fig. 5c and d).

**TABLE 1:** Autofluorescence emission values measured from the spermatophore capsule and sperm bundle expressed as means (a.u.), standard deviations, and standard errors^*a*^

Capsule or bundle	Mean	Std Dev	Std error	Significance
Spermatophore capsule				
Spermatophore N (*n* = 18)	2,184.11	1,588.67	374.45	NS
Spermatophore P (*n* = 35)	1,417.51	869.17	146.92	
Sperm bundle				
Sperm bundle N (*n* = 16)	1,718.24	1,450.25	362.56	*P* < 0.05
Sperm bundle P (*n* = 41)	872.73	731.95	147.09	

^*a*^ Data were calculated as integrated values of the 410–550 nm interval of each emission spectral area.

### *Spiroplasma* gene expression in testes and MAGs

To assess the potential presence of *Spiroplasma* transcripts in testes and MAGs, all the reads were mapped to the host *Gff* genome, searching for unmapped reads that could belong to the bacterium. It was found that 93% of reads mapped to the *Gff* genome (GCF_014805625.2), with a remaining proportion of unmapped reads (7%). These unmapped reads were mapped against the *s*Gff genome (22). Among these unmapped reads, only four genes had a mean read count greater than 10: ribosomal genes (23S and 16S rRNA), a transfer-messenger RNA (tmRNA; *ssrA*), and a ribonuclease (*RNaseP_bact_b*).

### *s*Gff and *Gff* microbiota interaction

A metataxonomic analysis was attempted, although our bulk transcriptomics data were not specifically suited for this purpose (see Materials and Methods): no rRNA depletion steps were performed prior to sequencing. Despite this limitation, a total of 338 genera of bacteria were retrieved in testes (mean abundance threshold across samples = 10). Considering bacterial genera having a minimum relative abundance of 0.1% in at least 3 out of 12 samples, 57 genera resulted significantly differentially present between infected and uninfected testes (Table 2). Among these, 49 genera had a significantly greater abundance in *s*Gff-infected, while 8 genera had a lower abundance. Among the most differentially abundant bacteria, in addition to *Spiroplasma* (l2FC = 2.89; baseMean = 30,616.42), were found *Wolbachia* (l2FC = 1.34; baseMean = 428.73) and *Wigglesworthia* (l2FC = 4.33; baseMean = 15,722.29). In MAGs, 263 genera were detected (mean abundance threshold across samples = 10), and no significant differences in genus abundance were found between infected and uninfected samples.

**TABLE 2:** List of the differentially abundant bacteria found in the transcriptomic data set

Genus	baseMean	l2FC	lfcSE	*P*adj	Gram coloration
*Spiroplasma*	30,616.42	2.89	1.00	0.04	Gram-neutral
*Wigglesworthia*	15,722.29	4.33	1.18	0.01	Gram-negative
*Romboutsia*	4,349.93	2.64	0.73	0.01	Gram-positive
*Buchnera*	3,595.05	4.40	1.18	0.01	Gram-negative
*Pseudomonas*	2,602.00	0.49	0.12	0.00	Gram-negative
*Sphingomonas*	1,474.43	−0.28	0.10	0.05	Gram-negative
Candidatus *Blochmanniella*	1,106.38	4.12	1.11	0.01	Unknown
*Wolbachia*	428.73	1.34	0.22	0.00	Gram-negative
*Dactylococcopsis*	258.92	0.80	0.27	0.03	Unknown
*Proteus*	213.58	3.80	0.97	0.01	Gram-negative
Candidatus *Riesia*	179.23	5.10	1.41	0.01	Unknown
*Xenorhabdus*	148.50	0.75	0.27	0.05	Gram-negative
*Dietzia*	144.11	4.00	1.10	0.01	Gram-positive
Candidatus *Profftia*	132.41	4.29	1.12	0.01	Unknown
*Brachybacterium*	125.31	3.18	1.04	0.03	Gram-positive
*Massilia*	120.52	−0.61	0.18	0.01	Gram-negative
*Colwellia*	114.81	4.82	1.30	0.01	Gram-negative
*Photorhabdus*	112.94	1.30	0.27	0.00	Gram-negative
*Erwinia*	111.14	2.45	0.80	0.03	Gram-negative
*Moraxella*	109.18	−0.68	0.24	0.04	Gram-negative
*Thalassotalea*	100.82	5.64	1.26	0.00	Gram-negative
*Chryseobacterium*	85.60	1.29	0.47	0.05	Gram-negative
Candidatus *Palibaumannia*	74.03	3.06	0.96	0.02	Unknown
*Providencia*	73.87	3.05	0.85	0.01	Gram-negative
*Leptotrichia*	68.53	−3.04	1.08	0.05	Gram-negative
*Prochlorococcus*	62.87	4.78	1.40	0.01	Gram-negative
Candidatus *Hamiltonella*	57.47	3.64	1.03	0.01	Unknown
*Mammaliicoccus*	42.05	5.62	1.46	0.01	Gram-positive
*Pectobacterium*	40.48	0.96	0.30	0.02	Gram-negative
*Psychrobacter*	40.13	1.68	0.60	0.05	Gram-negative
*Pseudoduganella*	32.98	−0.67	0.24	0.05	Gram-negative
*Citricoccus*	32.43	4.50	1.33	0.01	Gram-positive
*Nitrosomonas*	31.45	4.11	1.11	0.01	Gram-negative
*Turicibacter*	30.41	4.90	1.56	0.02	Gram-positive
*Rhodopseudomonas*	30.27	−0.90	0.29	0.02	Gram-negative
*Anaerostipes*	27.23	5.28	1.35	0.01	Gram-variable
*Paraphotobacterium*	26.78	5.14	1.54	0.01	Gram-negative
Candidatus *Purcelliella*	26.70	4.46	1.22	0.01	Unknown
*Algoriphagus*	25.54	1.00	0.33	0.03	Gram-negative
Candidatus *Tachikawaea*	24.74	4.74	1.35	0.01	Unknown
*Agarilytica*	23.41	5.65	1.39	0.00	Gram-negative
*Blattabacterium*	22.65	3.30	0.80	0.00	Gram-negative
*Geminocystis*	20.82	4.52	1.23	0.01	Unknown
Candidatus *Schneideria*	19.78	3.55	0.92	0.01	Unknown
*Actinobacillus*	17.53	4.25	1.24	0.01	Gram-negative
Candidatus *Mikella*	15.62	3.51	1.16	0.03	Unknown
*Francisella*	14.85	1.07	0.37	0.04	Gram-negative
Candidatus *Ishikawella*	13.48	5.35	1.43	0.01	Unknown
*Calothrix*	10.78	3.24	0.90	0.01	Unknown
*Metamycoplasma*	10.29	5.43	1.76	0.03	Gram-neutral
*Alysiella*	10.11	1.18	0.42	0.04	Gram-negative
*Niallia*	9.76	2.73	0.82	0.01	Gram-positive
*Avibacterium*	9.20	6.85	1.81	0.01	Gram-negative
*Asticcacaulis*	7.65	−1.31	0.45	0.04	Gram-negative
*Selenomonas*	7.55	−2.96	0.97	0.03	Gram-negative
*Gimesia*	5.67	2.28	0.78	0.04	Gram-negative
*Filifactor*	5.51	4.53	1.59	0.04	Gram-positive

## DISCUSSION

We investigated the impact of the *Spiroplasma* strain *s*Gff on gene expression in the reproductive tissues of its host, *Glossina fuscipes fuscipes*. To minimize noise from environmental variables, the study used a laboratory *Gff* strain. Since previous attempts to produce fully infected or uninfected lines did not yield 100% homogeneous results (35), we followed established protocols by verifying infection status of single males via PCR (23, 24, 26).

### Testes and MAGs gene transcription profiles reflect the different functions of these reproductive tissues

A greater number of genes were transcribed in testes compared to MAGs, but MAGs displayed an overall higher expression level. These results were expected, considering the different but complementary roles of these organs in *Glossina* male reproduction. Testes contribute to sperm composition and fertilization functions, while MAGs mainly contribute to semen composition of the ejaculate (7, 36, 37). While some of the MAG-biased genes encode unknown functions, others encode enzyme inhibitors and peptidase regulators typical of semen composition (7, 36, 37). On the other hand, testes-biased genes are associated with mitochondrial functions, binding, oxidoreductase/transferase activities, and cytoskeletal and lipid/carbohydrate transporter functions, which are more conserved than the MAG-biased genes among the different *Glossina*, *Drosophila,* and other insect taxa (7, 36). The high conservation observed for sperm-associated genes may reflect essential sperm-specific processes they participate in. In contrast, MAG genes encode key factors in species-specific male reproductive success and, as such, are subject to rapid evolution resulting from sexual conflict and competition that are very high in *Glossina* (7). Indeed, viviparity entails the production of only a few progeny (12), leading to conflict between competing individual males for achieving success in fertilization (38). In the *Gff* riverine species from the *Palpalis* subgroup, the selective pressure level in MAGs is higher than in savannah flies such as the *Morsitans* subgroup (7). *Gff* flies typically live in narrow riverine habitats and suffer seasonal demographic fluctuations. During the dry season, populations undergo a dramatic demographic contraction with the remaining flies concentrating in most refugia. At the end of the dry season, within the residual population emerging after the bottleneck, the strength of male competition increases because of the greater number of interactions for achieving copulation.

### *s*Gff differentially affects testes and MAGs

**Fig 6:**
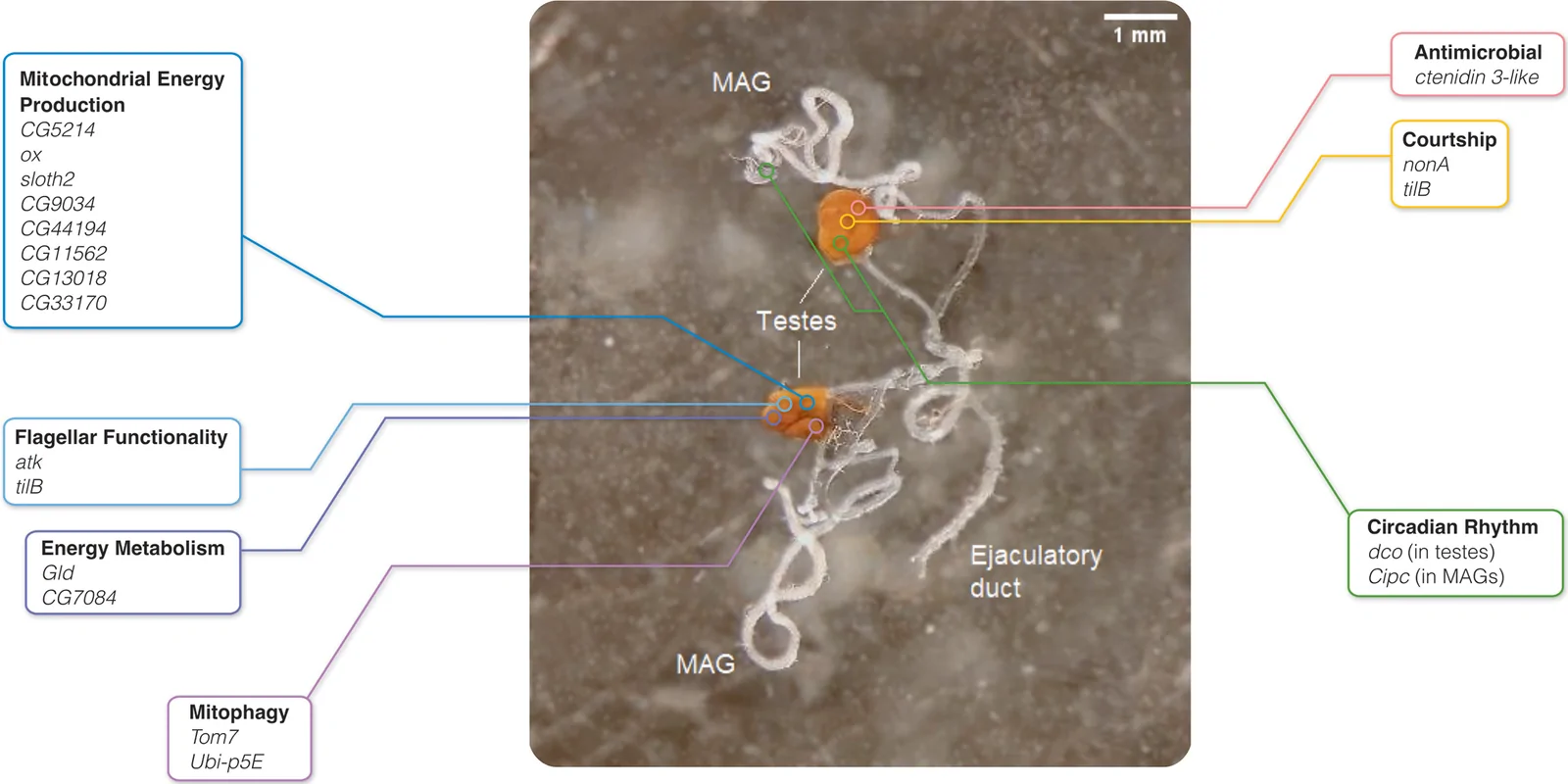
*Glossina fuscipes fuscipes* whole male reproductive tracts. In the boxes, differentially expressed genes are reported, discussed, and grouped by their products’ functionality. Inkscape v.1.4.2 was used to produce this schema. Reproductive tissue figure scale bar = 1 mm. MAG, male accessory gland.

Assuming the *Gff* orthologs have a similar function associated with the *D. melanogaster* or *G. morsitans* counterparts, *s*Gff differentially affected the two reproductive organs (Fig. 6). Indeed, it seems to marginally affect MAGs expression profile, as only one gene was affected. This gene is involved in circadian rhythm. On the other hand, *s*Gff altered the expression levels of 88 genes in testes, affecting genes involved in the circadian rhythm, energy metabolism, sperm functionality, and the microbial community. Notably, the abundance of *Spiroplasma* in MAGs (baseMean = 8,710 ± 3,585) is much lower than in testes (baseMean = 47,688 ± 29,820), which could explain such a difference in gene expression.

### *s*Gff may alter the circadian rhythm of reproductive tissues

The ortholog of *Drosophila Cipc* (Clock Interacting Protein Circadian) has been found upregulated in Gff-infected MAGs. In *Drosophila,* this gene encodes a protein with a conserved repressive function within the CLK-CYC transcriptional feedback loop, a key regulator of the circadian rhythm (39). Its upregulation is associated with lengthening of the circadian period, and it has been found to be expressed also in male semen-secreting cells (40). Considering the role of MAGs in semen production (7) and the fact that the mating behavior in *Gff* is tightly regulated by a circadian rhythm, with peaks in the morning and afternoon (41), it is tempting to hypothesize that in infected males, Cipc upregulation could putatively lengthen the rhythms, potentially also affecting the timing of semen production.

The ortholog of the *Drosophila* discs overgrown (*dco*), also known as Doubletime (DBT), was downregulated in testes in response to *s*Gff infection. *dco* encodes a kinase that phosphorylates PER (Period), targeting it for degradation, therefore representing a crucial component regulating circadian rhythm (38). However, when cytoplasmic levels of TIM (Timeless) are high, PER binds to TIM, forming a complex that represses the CLK-CYC system upon DBT binding (42). In *Drosophila*, DBT kinase reduction lengthens the circadian rhythm due to desynchronization of the clock network (42).

Assuming that *Gff* orthologs have the same functionality observed in *D. melanogaster*, the impact of *s*Gff on these genes suggests a putative downregulation of the circadian rhythm in both MAGs and testes, which, in turn, might affect reproductive fitness. Indeed, *per* and *tim* mutants in *D. melanogaster* exhibited reduced sperm production and altered sperm quality, as well as impaired maturation and release from the testes, ultimately resulting in reduced fertility (43). Additionally, the speculative rhythm alteration in *Gff* is further supported by the downregulation of *nonA* (l2Fc = −0.76), a sex-linked gene encoding an RNA-binding protein involved in visual behavior and courtship song of *D. melanogaster*. Mutation of *nonA* can disrupt the courtship song of flies (44). Another gene that was shown to affect song behavior in *Drosophila* was found upregulated: LOC119644721 (ortholog of *D. melanogaster* gene *tilB*; l2FC = 0.88) (45).

The differential expression of both circadian rhythm and courtship genes may be related to the altered mating behavior previously observed in *Gff* in relation to *s*Gff infection (23, 35). However, since direct behavioral or physiological assays monitoring circadian cycles were not performed in *Gff*, further investigation is strictly required to validate this transcriptional hypothesis and determine whether these changes translate into actual phenotypic rhythm disruptions.

### *s*Gff has a negative impact on energy metabolism in testes and sperm

The *D. melanogaster* ortholog gene *tilB*, upregulated in testes, is not only involved in courtship as previously described but also affects flagellar function. This gene encodes a protein essential for the transport and/or assembly of the cilium/flagellum, and alterations in its expression can lead to sperm flagella dysfunction in *D. melanogaster* (29). Conversely, LOC119640098, an ortholog of the *D. melanogaster* gene *atk*, was downregulated. The *atk* gene encodes a protein that surrounds the cilium and is critical for its assembly, structural integrity, chemosensation, and proprioception (30), and it has also been positively associated with sperm viability in *Apis mellifera* (46).

Genes that display orthology with *D. melanogaster* genes associated with energy metabolism were negatively affected by *s*Gff. This is the case of *Gld* (l2FC = −0.62), which encodes glucose 1-dehydrogenase, a key enzyme in an alternative glucose metabolic pathway. Additionally, *CG7084* (l2FC = −1.85), which encodes a transmembrane cation transporter orthologous to the human solute carrier family 22 members 1–5 (SLC22A1–5), was downregulated. Notably, SLC22A5 encodes the organic cation/carnitine transporter 2, which in humans and mice is the main transporter involved in carnitine uptake in reproductive organs, and mutations in this gene were linked to male infertility (47). Carnitine plays a central role in energy metabolism (26) by shuttling long-chain fatty acids into mitochondria for β-oxidation, which produces ATP. Reduced carnitine transport could impair β-oxidation, leading to decreased energy availability for spermatogenesis and sperm function.

Mitochondrial energy production appears to be significantly disrupted by *s*Gff infection. Several genes critical to the citric acid (TCA) cycle and the electron transport chain (ETC) were downregulated. For instance, *CG5214* encodes dihydrolipoamide S-succinyltransferase, part of the 2-oxoglutarate dehydrogenase complex (48). Similarly, multiple genes involved in the ETC were downregulated, such as *ox* (l2FC = −1.10), which encodes ubiquinol-cytochrome c reductase subunit 9, a core component of complex III of the ETC (cytochrome c reductase). Another gene, *CG42308* (or *sloth2*), a component of complex III, was found to be downregulated (l2FC = −0.72). This enzyme facilitates electron transfer and proton translocation, playing a central role in oxidative phosphorylation and ATP production (31). *CG9034* (l2FC = −0.72) encodes NADH-ubiquinone oxidoreductase subunit A3, a key part of complex I of the ETC (NADH dehydrogenase) (49, 50). Additionally, genes involved in the assembly of cytochrome c oxidase (complex IV of the ETC), including *CG44194* (l2FC = −1.73), *CG11562* (l2FC = −1.34), *CG13018* (l2FC = −0.64), and *CG33170* (l2FC = −0.54), were also downregulated. Complex IV is essential for oxidative phosphorylation, and its proper function is crucial for spermatogenesis, sperm motility, and viability (51).

Mitophagy-related genes were also negatively impacted, suggesting that *s*Gff infection compromises mitochondrial homeostasis. This is the case of *Tom7* (l2FC = −0.82), a gene encoding a mitophagy and mitochondrial fission regulator (52–54). As well, two genes encoding parts of the ubiquitin-proteasome system, which is involved in mitophagy and the removal of damaged proteins (53, 55), were differentially expressed: *Ubi-p5E* (l2FC = 1.07), encoding ubiquitin-5E, and *RpL40* (l2FC = −0.59) (32).

The negative effects of *s*Gff on genes associated with sperm flagella, energy metabolism, and mitochondrial dynamics can be considered in light of the fact that *s*Gff is able to alter metabolic and reproductive homeostasis (24, 55). It competes for essential energetic substrates, thereby reducing their availability to testis cells (24). This competition may diminish mitochondrial activity and energy production (24, 26), ultimately impairing spermatogenesis and sperm quality by disrupting the expression of critical genes.

To rigorously validate this metabolic hypothesis, we employed a highly sensitive NADH autofluorescence assay, a powerful and non-invasive tool widely recognized for accurately profiling cellular energy states. It showed that sperm from *s*Gff-infected *Gff* had lower levels of NADH, thus reducing energy availability and lower mitochondrial functionality in *s*Gff-infected sperm (56). It is noteworthy that sperm from *s*Gff-infected males display a reduced beat frequency, suggesting reduced sperm motility (24). Taken together with the results on the negative impact on the expression of genes involved in energy metabolism, the reduced NADH levels and decreased beat frequency suggest that *s*Gff infection limits the energy available to sperm, leading to impaired function and competitiveness.

### *Spiroplasma* facilitates colonization of other bacterial species in testes

Unfortunately, we could not detect valuable *s*Gff gene expression information in reproductive tissues. However, despite limitations (see Materials and Methods), our metataxonomic analysis provides exploratory results and intriguing preliminary insights that warrant further validation. It revealed that, in *s*Gff-infected testes, 49 bacterial genera increased in abundance compared to *s*Gff-uninfected testes, while only 8 genera showed a decrease. Notably, *Spiroplasma* had the highest mean abundance across all samples (baseMean > 30 k), confirming that sGff is the primary bacterium distinguishing infected and uninfected samples, with no other bacteria showing comparable abundance. Notably, since the pipeline is based on the Kraken2 database, it is likely that some of the identified genera are actually the closest relatives of the real ones. However, we were able to retrieve, in such entries, very important genera for *Glossina*, such as *Wigglesworthia*, *Sodalis,* and *Wolbachia*.

Considering that *s*Gff induces the downregulation of *ctenidin-3-like*, a bacteriostatic agent against gram-negative bacteria and with reduced growth-rate activity against gram-positives, we can hypothesize that *s*Gff can affect the antimicrobial action in testes (36). On the other hand, *s*Gff is a wall-deficient bacterium, and as such, it should not be affected by the antimicrobial activity of *ctenidin-3-like*. As a result, less-dominant bacteria, and particularly gram-negative bacteria, may proliferate in sGff-infected testes and increase in density. Among them, *Wigglesworthia* is an obligate gram-negative mutualist hosted in the bacteriome and milk glands (57). It is noteworthy that *Wigglesworthia*, in addition to providing vitamin B6 to the host, interacts with the *Glossina* immune system, influencing the tsetse fly's ability to transmit trypanosomes (8, 10, 58, 59). We can thus suggest that the increase in refractoriness to the trypanosome observed in the presence of *s*Gff may be an effect of microbiota shifts also mediated by the increase in prevalence of *Wigglesworthia*.

Another bacterial genus with increased abundance in *s*Gff-infected testes was *Wolbachia*, an intracellular parasite that is much more abundant in *Glossina* species where *Spiroplasma* is not present (60). In *Gff*, its density and prevalence were found to be very low, confirmed by our results (average baseMean = 428.73; Table 2). However, it remains unclear whether *ctenidin-3-like* products act on intracellular parasites like *Wolbachia*; therefore, we cannot conclude that the increased abundance of *Wolbachia* is linked to reduced *ctenidin-3-like* expression.

### Conclusion

Here, we provide evidence that the *Spiroplasma* strain *s*Gff infection in *Glossina fuscipes fuscipes* leads to measurable transcriptional impacts on testicular function, reducing energy metabolism and sperm quality and interfering with circadian rhythms, probably impairing mating behavior. On the other hand, this bacterium does not seem to affect the functionality of MAGs, which are involved in semen production, a critical component for male reproductive success, but it uses the ejaculate as a way to further increase its transmission. It was previously demonstrated that *s*Gff infection in *G. f. fuscipes* influences male mating behavior with partner selection (23, 26), suggesting a form of infection-based non-random mating. All these findings underline how this bacterium can shape reproductive physiology, and consequently, it may shape the population structure of tsetse flies.

Another important question that deserves attention is the nature of the symbiotic relationship characterizing *s*Gff and *G. f. fuscipes*. With respect to host-microbiome interactions, previous results indicated a refractory effect against *Trypanosoma* (18), and our exploratory analysis further suggests that *s*Gff may positively influence *Wigglesworthia* abundance. Conversely, previous studies reported that *s*Gff compromises sperm fitness (24). Consistently, our results demonstrate a significant energetic impairment of the male reproductive organs. Cumulatively, these findings indicate that *s*Gff confers certain benefits but also imposes substantial costs to the host, keeping open the biological question of why this bacterium is maintained in this species despite not being present in all populations.

Based on all these considerations, we can propose that the data presented here not only add a valuable layer to further interpret the ecological and evolutionary narrative of *s*Gff interactions, but also underscore the bacterium’s potential as a candidate symbiont-based biocontrol agent. Although our data are derived from a specific laboratory strain, the intriguing modulation of the fly's reproductive fitness (23, 26) and the possible contribution to refractoriness to trypanosome infection (18) found let us hypothesize that *s*Gff could be strategically integrated into environmentally sustainable vector management programs, potentially in concert with the Sterile Insect Technique.

A final consideration regarding our data is that they were derived from a laboratory line that is maintained under stable conditions. Given that natural populations of *G. f. fuscipes* are genetically heterogeneous and subjected to seasonal fluctuation, further analyses across different developmental times and wild genetic backgrounds are needed to verify whether the presence of *s*Gff and its effect on its host are constrained by this seasonal variation (18) and to determine how effectively these laboratory-based findings can be generalized to field interventions.

## MATERIALS AND METHODS

### *Gff* strain and rearing conditions

Tsetse adults used in this study were obtained from the *Gff* colony maintained at the Joint FAO/IAEA Center, Insect Pest Control Laboratory (IPCL) in Seibersdorf, Austria. This colony was established in 1986 using flies collected from the Bouar Province, Central African Republic. Experimental flies were reared at 24°C ± 0.5°C and 75% ± 5% relative humidity. Before adult eclosion, pupae were kept separated, and as soon as adults emerged, males were separated from females. Each newly emerged male was screened for *s*Gff infection through careful surgical removal of one intermediate leg, performed under sterile conditions. Between one specimen and the next, both forceps and scissors, used for the leg excision, were sterilized through brief immersion in 70% EtOH followed by exposure to the flame of a Bunsen burner. The excised leg of each specimen was collected in a new tube, and the respective fly was placed in an individual fly-holding cage. Then, the excised legs were used for *s*Gff infection detection via PCR. Each male was maintained singularly to avoid cross-contamination between individuals until reproductive tissue dissection on the sixth day. Adult flies were fed every 2 days for 10–15 min on defibrinated bovine blood using a silicone membrane (61). The first blood feeding occurred the day after eclosion, once *s*Gff infection status was assessed to allow the separation of *s*Gff-infected flies from *s*Gff-uninfected flies during blood feeding.

### Determination of the infection status

DNA extraction on the excised legs was performed using the ZR *Quick*-DNA 96 kit (Zymo Research, California, USA). A multiplex PCR using two specific primer pairs was used to assess the infection status of the adult males and verify the quality of the extracted DNA: Spi16s primers (F: 5′-GGGTGAGTAACACGTATCT-3′, R: 5′-CCTTCCTCTAGCTTACACTA-3′), a *s*Gff-specific primer pair allowed the identification of the bacterial infection, and a *Gff*-specific primer pair amplifying the housekeeping gene ß-tubulin (F: 5′-ACGTATTCATTTCCCTTTGG-3′, R: 5′-AATGGCTGTGGTGTTGGACAAC-3′) (24). The PCR was performed using the following cycling conditions: 94°C for 5 min, 34 cycles of 94°C for 45 s, 58°C for 45 s, 72°C for 1 min, and a final extension at 72°C for 10 min. Amplification products were electrophoresed on 2% agarose E-Gel (Fischer Scientific) stained with SYBR Safe.

### Dissections and RNA-seq sample preparation

Six-day-old virgin males were dissected under a stereomicroscope in sterile 1× phosphate-buffered saline (PBS), and for each male, testes and MAGs were collected; dissections were conducted carefully, and all the fat body surrounding the reproductive tract was removed. For both *s*Gff-infected and *s*Gff-uninfected, 6 pools of testes and 6 pools of MAGs were obtained, each pool containing tissues collected from 10 males. Immediately after dissection, the dissected tissues were put into a sterile tube containing ice-cold 500 μL of TRIzol Reagent (Thermo Fisher Scientific), and once the pools were complete, tissues were pestled and stored at −80°C.

### RNA extraction and library preparation

The resulting 24 samples were shipped to Edinburgh Genetics Ltd for RNA extraction and quality control (QC) assessment. All 24 samples passed the QC process. The library construction was carried out following Illumina standard protocols, and total RNA sequencing was performed.

### Differential gene expression analysis

The paired-end sequences from testes and MAGs of *s*Gff-infected and *s*Gff-uninfected tsetse flies were analyzed through the nf-core/rnaseq pipeline version 3.12.0 (62, 63). Briefly, the pipeline consists of four steps: pre-processing, where raw reads are controlled and filtered; genome alignment and quantification; post-processing, where mapped reads are sorted, indexed, deduplicated, and transcripts are assembled and quantified; finally, a multiQC file (64) is produced, which summarizes results obtained from the pipeline. The STAR-Salmon method was employed, which uses STAR (65) for the genome alignment and Salmon (66) for the expression quantification. The alignment was performed against the IAEA *Gff*-annotated genome (67) (version 63 of VectorBase, GCF_014805625.2 in NCBI). All the annotated proteins of the reference genome were blasted against the *D. melanogaster* database, wrapping BLASTP through the NcbiblastpCommandline module from Biopython version 1.81 (68), to retrieve the most likely *D. melanogaster* ortholog gene symbols for each annotated protein.

The count matrix produced by the rnaseq pipeline was used to perform the gene expression analysis in R version 4.2.3 (69). PCA was performed using plotPCA from the DESeq2 package (70, 71). Only genes with a mean read count of at least 10 were considered. DEGs were detected employing the DESeq2 package as well, considering adjusted *P*-value ≤0.05 and |l2FC| ≥2 when comparing testes and MAGs. Adjusted *P*-value ≤ 0.05 and a more relaxed |l2FC| (≥0.5, equivalent to an approximate fold change of 1.42 times) were adopted when comparing infected and uninfected samples. This choice was made to capture possible subtle but biologically meaningful transcriptional modulations where minor shifts in expression can lead to downstream phenotypic effects. Afterward, DEGs were employed for ORA using the clusterProfiler package version 4.9.0 (72) and *Gff* gene ontology (GO) terms provided in the GAF file downloaded from NCBI (GCF_014805625.2_Yale_Gfus_2_genomic.gaf) integrated with the *D. melanogaster* GO terms for orthologous genes. biomaRt (73, 74) was employed to ease the translation of gene symbols into gene identifiers. Plots were generated using ggplot2 (75).

Metabolic pathways affected by *s*Gff were detected using STRING (33). DEGs having a *D. melanogaster* ortholog were used for this analysis, as *D. melanogaster* served as the reference organism. Afterward, connected networks were selected, and metabolic pathways associated with them were identified by inspecting KEGG and Reactome enrichment results.

GO terms and metabolic pathways affected by *s*Gff were further investigated by performing a co-expression network analysis using the WGCNA package version 1.73, followed by ORA on subnetworks containing DEGs (76, 77).

### Microbiota analysis

After mapping the reads to the *Gff* genome (78), any unmapped reads were subsequently remapped to the *s*Gff genome (22) using the nf-core/rnaseq pipeline, with settings carefully tuned for bacterial genomes, as recommended in the user manual.

Metataxonomic profiling of the reads was performed using the nf-core/hgtseq pipeline (62, 63, 79). Briefly, unmapped reads were classified using Kraken2 (80–82), and the metataxonomic profiles of each sample were stored in a TSV file. The Kraken2 database was constructed by supplementing the standard database with all available reference genomes of *Mollicutes* (*s*Gff class; Table S7) and the genome of *Gff*. The hgtseq pipeline also flagged likely contaminant taxa (79), which were excluded from subsequent analyses. An additional decontamination step, based on a frequency approach, was carried out in R using the decontam package (83). Briefly, the likelihood of a taxon being a contaminant was estimated based on its frequency across samples. Finally, phyloseq and DESeq2 (70, 71) were used to identify bacteria that were differentially present in relation to *s*Gff infection. Gram coloration was assessed through BacDive (84). In cases of no results, it was considered the gram coloration of the closest relative with this information available in the database or searched in PubMed. Candidatus genera were declared “Unknown.”

Notably, we attempted to perform a metataxonomic analysis on our bulk transcriptomics samples that were not depleted for rRNA prior to sequencing, and sequencing depth was insufficient to accurately capture bacterial transcriptomes (85). Consequently, when aligning unmapped reads to the sGff genome, only rRNA genes were recovered, consistent with the fact that prokaryotic rRNA is vastly more abundant than mRNA (85–88). Without rRNA depletion or substantially deeper sequencing, the imbalance in RNA species severely limits the retrieval of bacterial mRNA (85). Moreover, the Kraken2 database used for metataxonomic profiling included only bacterial species with previously documented interactions or associations with Gff. This constraint inevitably excluded taxa that may have been present in the samples but were not represented in the reference database (89). Additional limitations arose during decontamination, as no negative controls were available to apply a “presence-based” contaminant removal strategy (83). Therefore, only taxa flagged as contaminants by hgtseq (79) or identified using the frequency-based method implemented in decontam (83) could be removed.

### Autofluorescence assay on *Gff* sperm bundle

NADH autofluorescence imaging was used for visualizing changes in energy availability between *s*Gff-infected and -uninfected sperm. A mating experiment was performed using virgin *Gff* 8-day-old females and 6-day-old males. At the end of the copula, when individuals spontaneously separated, the spermatophore was immediately dissected from the female’s uterus to avoid the dissolution of the spermatophore and the migration of the spermatozoa in the spermathecae (37). Dissections were conducted under a stereomicroscope (Leica MZ12) in sterile PBS 1×, and the sperm bundle was isolated from the spermatophore capsule; for each mating pair, the legs of the males were collected for the determination of the infection status. The sperm bundle was transferred to a clean microscope glass slide, previously washed with a 50% alcohol-ether solution, and allowed to dry at room temperature. The spermatophore capsule was subjected to the same treatment. The microscope slides were then stored in the dark at room temperature until the autofluorescence assay. Twenty samples of sperm bundles and the relative spermatophore capsule were collected and analyzed. Genomic DNA was extracted from the excised male legs using the protocol described in Baruffi et al. (90), and subsequently, a PCR with *s*Gff-specific primers was performed, as described before. AF spectroscopy was performed at IGM-CNR (Pavia, Italy) on unfixed, unstained sperms under epi-illumination, using a fluorescence microscope (Leitz, Wetzlar, Germany) integrated with a PMA 12 Optical Multichannel Analyzer (Hamamatsu Photonics Italia s.r.l., Arese, MI). The light excitation source was an LED-366 nm (Hamamatsu Photonics) with two separate optical fibers: one to guide the excitation light to the samples, and another to collect the autofluorescence emission for the measuring system. Excitation and emission light beams have been selected through heat-absorbing filters (KG1-BG38) and interferential (366 nm, *T*_366_ = 25%) filters, a dichroic mirror (390 nm, *T*_390_ < 2%) and a long pass filter (>420 nm). Spectra were collected in the 400–750 nm interval.

### Statistical analysis

R version 4.2.3 (69) was used to perform all statistical analyses. To assess the significance of comparisons concerning tissues and sGff infection status, the Mann-Whitney *U* test (91) was employed for single or pairwise comparisons and the Kruskal-Wallis test for multiple comparisons, using the rstatix package (92). In case of multiple pairwise comparisons, Benjamini-Hochberg *P*-value correction was employed (93).

## Data Availability

Sequencing data are available on NCBI under the PRJNA1381280 project, accession IDs SAMN54103317–SAMN54103340. Code used for the analysis in R is available at https://github.com/RiccPicc/gff_sgff_rnaseq_testes_mags.

## SUPPLEMENTAL MATERIAL

Supplemental material msystems.00526-26-s0001.pdf Fig. S1-S4; supplemental table captions.

Table S1 msystems.00526-26-s0002.xlsx Differentially expressed genes between testes and male accessory glands.

Table S2 msystems.00526-26-s0003.xlsx Results of the over-representation analysis based on testis versus male accessory gland differential gene expression.

Table S3 msystems.00526-26-s0004.xlsx Differentially expressed genes between *Spiroplasma*-infected and -uninfected male accessory glands.

Table S4 msystems.00526-26-s0005.xlsx Differentially expressed genes between *Spiroplasma*-infected and -uninfected testes.

Table S5 msystems.00526-26-s0006.xlsx GO terms associated with the co-expression network built on the differentially expressed genes between *Spiroplasma*-infected and -uninfected testes.

Table S6 msystems.00526-26-s0007.xlsx GO, KEGG, and Reactome terms associated with STRING network built on the differentially expressed genes between *Spiroplasma*-infected and -uninfected testes.

Table S7 msystems.00526-26-s0008.xlsx Bacterial genomes added in Kraken2 database.

ASM does not own the copyrights to Supplemental Material that may be linked to, or accessed through, an article. The authors have granted ASM a non-exclusive, world-wide license to publish the Supplemental Material files. Please contact the corresponding author directly for reuse.
