# Supplementary material for: Spiroplasma in Glossina fuscipes fuscipes: a potential manipulator of male reproduction
Source: mSystems. 2026 Sep 11;11(10):e00526-26. doi: 10.1128/msystems.00526-26 (PMC13712805; doi:10.1128/msystems.00526-26)
Supplement: Supplementary file 1 [file msystems.00526-26-s0001.pdf]

Spiroplasma in *Glossina fuscipes fuscipes*: a manipulator of male reproduction

Riccardo Piccinno[a], Giulia Fiorenza[a], Francesco Lescai[a], Simone Carpanzano[a], Fabian Gstöttenmayer[b,c], Kiswend-sida M. Dera[c], Anna Cleta Croce[d], Chantel J. de Beer[c], Brian L. Weiss[b], Mariangela Santorsola[a], Alberta Pinnola[a], Giuliano Gasperi[a,d], Federico Forneris[a,e], Serap Aksoy[b], Adly M. M. Abd-Alla[c]#, Anna Rodolfa Malacrida[a]#

[a] Department of Biology and Biotechnology "Lazzaro Spallanzani", University of Pavia, 27100 Pavia, Italy

[b] Department of Epidemiology of Microbial Diseases, Yale School of Public Health, New Haven, CT 06510, United States of America

[c] Insect Pest Control Laboratory, Joint FAO/IAEA Programme of Nuclear Techniques in Food and Agriculture, 1400 Vienna, Austria

[d] Institute of Molecular Genetics (IGM), Italian National Research Council (CNR), 27100 Pavia, Italy

[e] Fondazione IRCCS Policlinico San Matteo, Pavia, Italy

Running title: *Spiroplasma* impact on tsetse fly reproduction

# Address correspondence to annarodolfa.malacrida@unipv.it and A.M.M.Abd-Alla@iaea.org

Keywords: *Spiroplasma, Glossina fuscipes fuscipes*, microbiota, male reproduction, energy metabolism, differential gene expression

Supplementary Figures

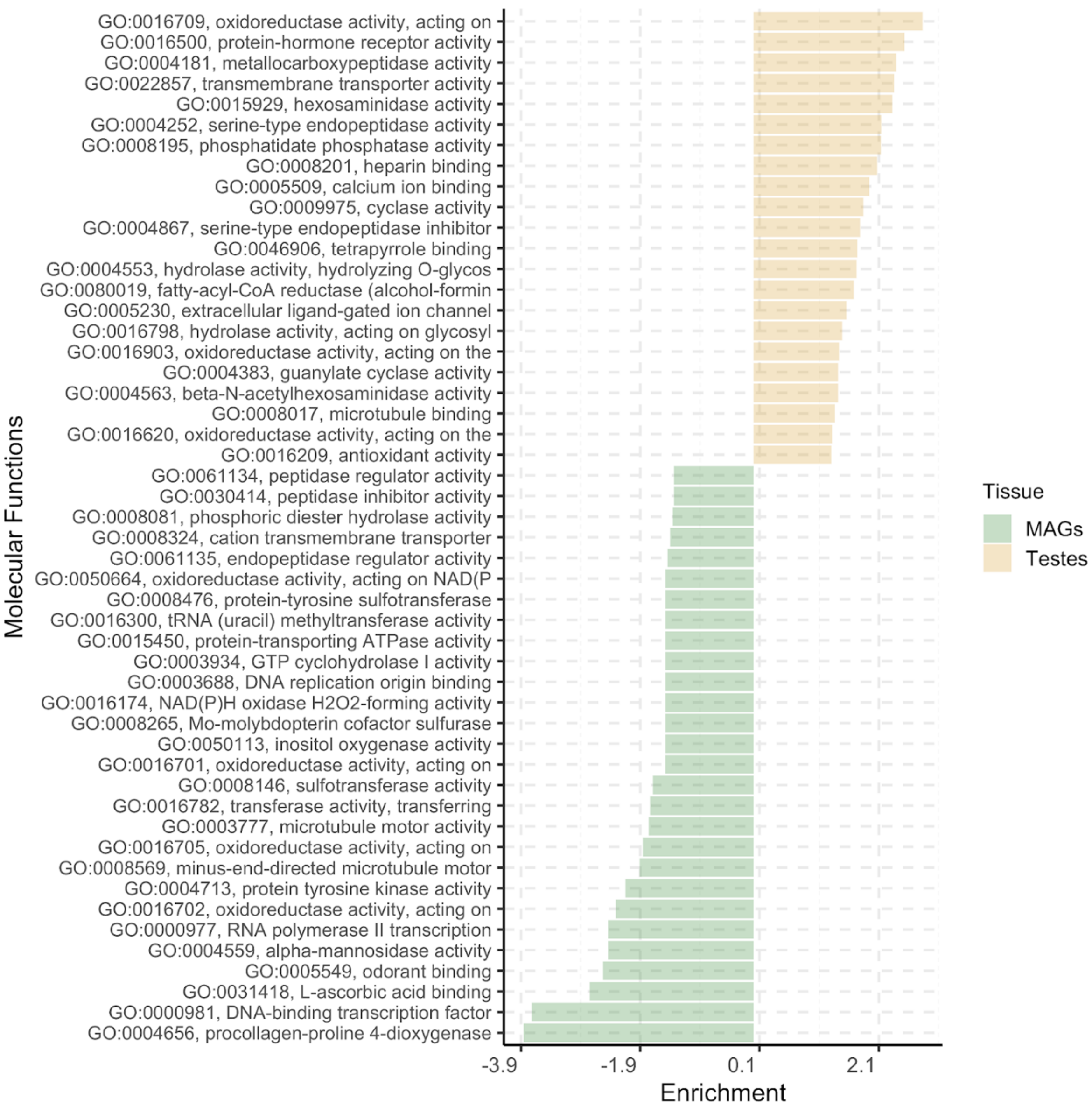

Fig. S1 Gene set enrichment analysis results considering molecular function GO terms of *Glossina fuscipes fuscipes* genome annotations. x-axis reports Enrichment value, which is the elimination of the Kolmogorov-Smirnov (KS) statistics provided by topGO singed based on the gene set for which it was tested (positive values refer to testes-biased DEGs, negative values to MAGs-biased DEGs). Only uninfected samples were considered for this analysis in order to have a picture of the gene expression profile of Gff testes and MAGs without possible effects due to *s*Gff presence.

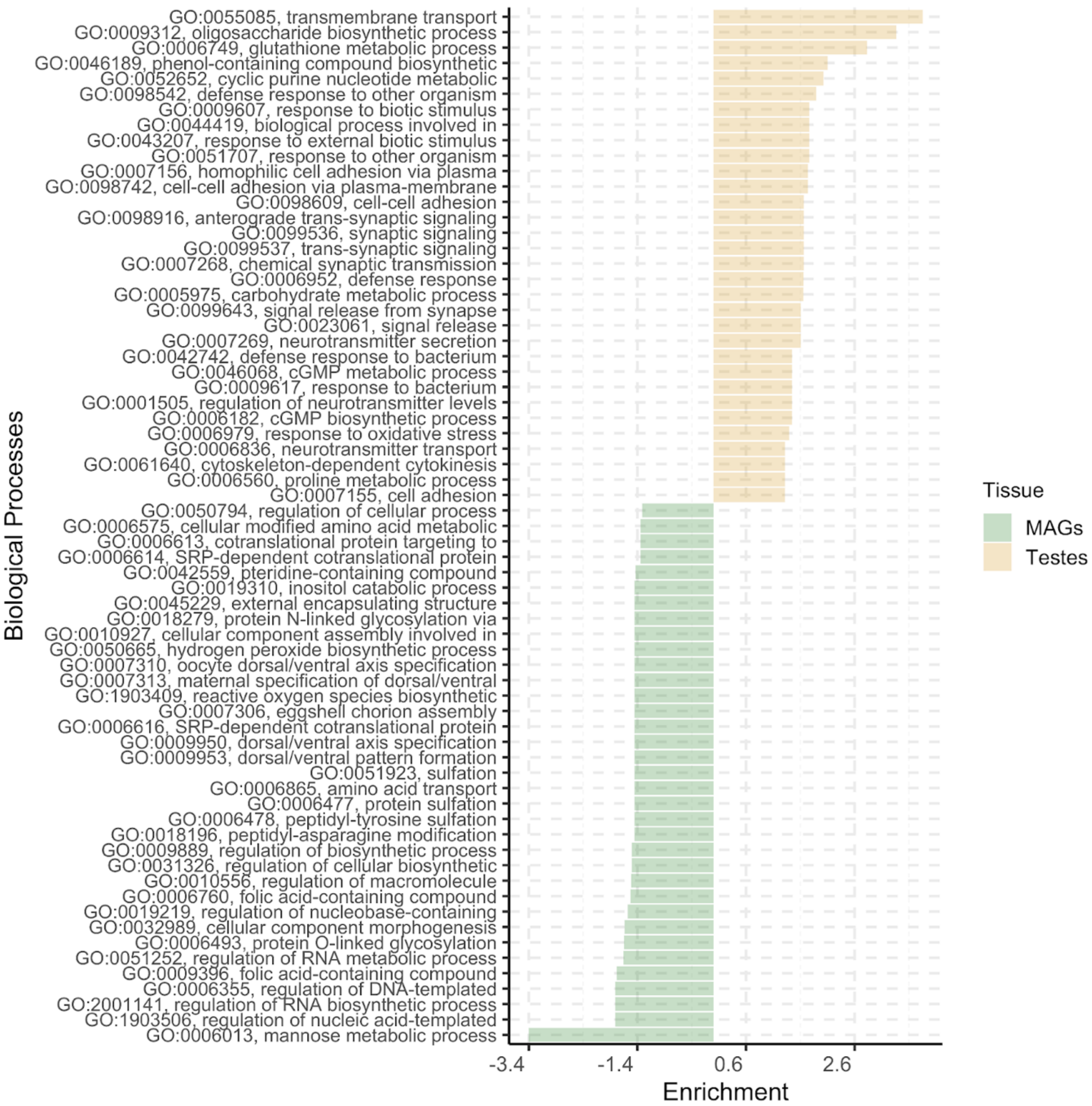

Fig. S2 Gene set enrichment analysis results considering biological processes GO terms of *Glossina fuscipes fuscipes* genome annotations. x-axis reports Enrichment value, which is the elimination of the Kolmogorov-Smirnov (KS) statistics provided by topGO singed based on the gene set for which it was tested (positive values refer to testes-biased DEGs, negative values to MAGs-biased DEGs). Only uninfected samples were considered for this analysis in order to have a picture of the gene expression profile of Gff testes and MAGs without possible effects due to *s*Gff presence.

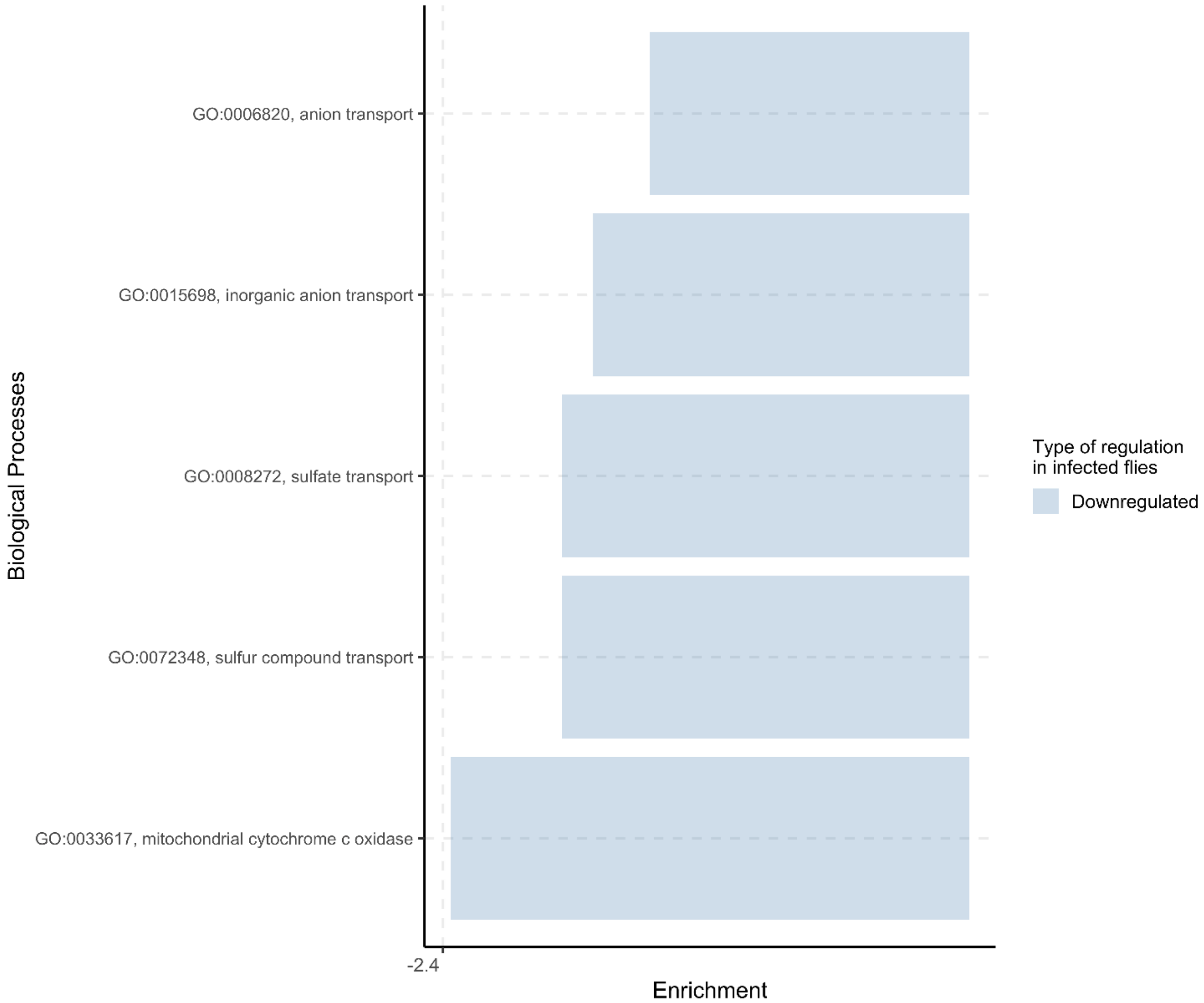

Fig. S3 Gene set enrichment analysis results considering biological processes GO terms of *Glossina fuscipes fuscipes* genome annotations. x-axis reports Enrichment value, which is the elimination of the Kolmogorov-Smirnov (KS) statistics provided by topGO singed based on the gene set for which it was tested. All values were negative, referring to downregulated genes in *s*Gff infected samples.

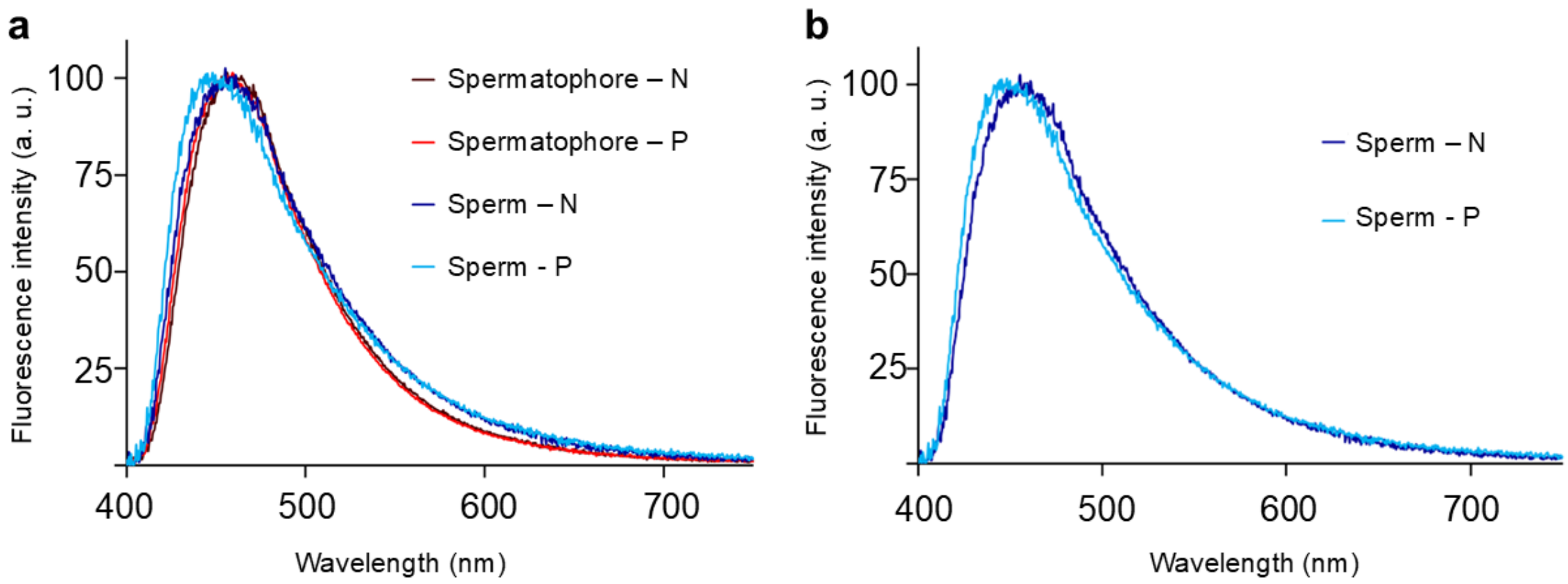

Fig. S4 The curves represent the mean autofluorescence spectra from spermatophore and sperm bundle samples (**A**) collected from *s*Gff-uninfected (N) or *s*Gff-infected (P) individuals. (**B**) To better appreciate the slight blue shift of the emission from P compared to N spermatozoa, their emission spectra are shown also separately from those of the spermatophores. All spectra are normalized to the maximum peak value of 100 a.u. (fluorescence intensity) to facilitate the comparison of the emission profiles.

Supplementary Tables

Table S1 List of all the genes transcribed in uninfected samples considering the expression cutoff value of 10 according to DESeq2. Negative log2FoldChange values indicate MAGs-biased genes. Positive log2FoldChange values indicate testes-biased genes. MAG = male accessory glands.

Table S2 Gene ontology terms found performing an over-representation analysis considering the differentially expressed genes found comparing uninfected MAGs and testes. Negative enrichment values indicate MAGs-biased genes. Positive enrichment values indicate testes-biased genes. MAG = male accessory glands, BP = Biological Process, MF = Molecular Function, CC = Cellular Component.

Table S3 List of all the genes transcribed in MAGs samples considering the expression cutoff value of 10 according to DESeq2. Negative log2FoldChange values indicate genes under-expressed in infected samples. Positive log2FoldChange values indicate genes over-expressed in infected samples. MAG = male accessory glands.

Table S4 List of all the genes transcribed in testes samples considering the expression cutoff value of 10 according to DESeq2. Negative log2FoldChange values indicate genes under-expressed in infected samples. Positive log2FoldChange values indicate genes over-expressed in infected samples. MAG = male accessory glands.

Table S5 Significant GO terms, Reactome and KEGG pathways found performing an over-representation analysis considering the differentially expressed genes found comparing uninfected and infected testes. MAG = male accessory glands, BP = Biological Process, MF = Molecular Function, CC = Cellular Component, GO = Gene Ontology.

Table S6 Significant GO terms, Reactome and KEGG pathways found performing an over-representation analysis considering the co-expression network containing most of the differentially expressed genes found comparing uninfected and infected testes. MAG = male accessory glands, BP = Biological Process, MF = Molecular Function, CC = Cellular Component, GO = Gene

Table S7 List of the species and relative NCBI taxid that were included in the Kraken2 database to perform the metataxonomy analysis with hgtseq nf-core pipeline.
